# 1,3-Dimethyl­benzo[*b*]dibenzothio­phene

**DOI:** 10.1107/S1600536809003456

**Published:** 2009-01-31

**Authors:** P. R. Umarani, A. Marx, V. Dhayalan, A. K. Mohanakrishnan, V. Manivannan

**Affiliations:** a Department of Physics, Presidency College, Chennai 600 005, India; b Department of Organic Chemistry, University of Madras, Guindy Campus, Chennai 600 025, India

## Abstract

The molecule of the title compound, C_18_H_14_S, is approximately planar (r.m.s. deviation = 0.029 Å). The crystal packing is stabilized by weak inter­molecular C—H⋯π inter­actions.

## Related literature

For the pharmacological activities of thio­phen derivatives, see: Dzhurayev *et al.* (1992 ▶); El-Maghraby *et al.* (1984 ▶); Gewald *et al.* (1996 ▶). For related structures, see: Harrison *et al.* (2006 ▶); Palani *et al.* (2006 ▶). For bond-length data, see: Allen *et al.* (1987 ▶).
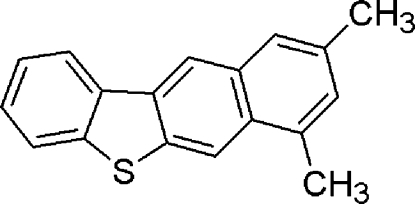


## Experimental

### 

#### Crystal data

C_18_H_14_S
*M* *_r_* = 262.35
Monoclinic, 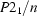

*a* = 10.0219 (3) Å
*b* = 5.8692 (5) Å
*c* = 22.8554 (5) Å
β = 99.787 (1)°
*V* = 1324.80 (12) Å^3^
*Z* = 4
Mo *K*α radiation
μ = 0.23 mm^−1^
*T* = 295 (2) K
0.26 × 0.20 × 0.18 mm

#### Data collection

Bruker Kappa APEXII diffractometer
Absorption correction: multi-scan (*SADABS*; Sheldrick, 1996 ▶) *T*_min_ = 0.944, *T*_max_ = 0.961
27929 measured reflections
3030 independent reflections
2574 reflections with *I* > 2σ(*I*)
*R*_int_ = 0.029

#### Refinement

*R*[*F* ^2^ > 2σ(*F* ^2^)] = 0.046
*wR*(*F* ^2^) = 0.137
*S* = 1.08
3030 reflections
174 parameters
H-atom parameters constrained
Δρ_max_ = 0.26 e Å^−3^
Δρ_min_ = −0.29 e Å^−3^

### 

Data collection: *APEX2* (Bruker, 2004 ▶); cell refinement: *SAINT* (Bruker, 2004 ▶); data reduction: *SAINT*; program(s) used to solve structure: *SHELXS97* (Sheldrick, 2008 ▶); program(s) used to refine structure: *SHELXL97* (Sheldrick, 2008 ▶); molecular graphics: *PLATON* (Spek, 2003 ▶); software used to prepare material for publication: *SHELXL97*.

## Supplementary Material

Crystal structure: contains datablocks I, global. DOI: 10.1107/S1600536809003456/bt2861sup1.cif

Structure factors: contains datablocks I. DOI: 10.1107/S1600536809003456/bt2861Isup2.hkl

Additional supplementary materials:  crystallographic information; 3D view; checkCIF report

## Figures and Tables

**Table 1:** Hydrogen-bond geometry (Å, °) *Cg*1 is the centroid of the C7–C9/C14–C16 ring and *Cg*2 is the centroid of the C1–C6 ring.

*D*—H⋯*A*	*D*—H	H⋯*A*	*D*⋯*A*	*D*—H⋯*A*
C17—H17*C*⋯*Cg*1^i^	0.96	2.68	3.486 (2)	142
C18—H18*A*⋯*Cg*2^ii^	0.96	2.75	3.649 (3)	155

Symmetry codes: (i) 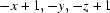; (ii) 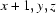.

**Fig. 1.**
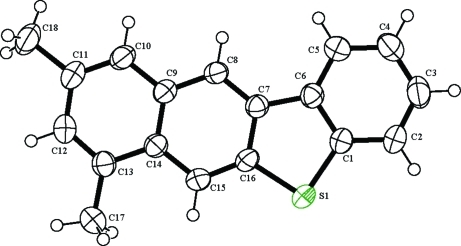
The molecular structure of (I), with atom labels and 50% probability displacement ellipsoids for non-H atoms.

## supplementary crystallographic information

### Comment

Thiophen derivatives possess pharmacological activities such as anti-bacterial, anti-cancer, anti-inflammatory (El-Maghraby *et al.*, 1984; Dzhurayev *et al.*, 1992) and anti-toxic properties (Gewald *et al.*, 1996).

The geometric parameters of the title molecule (Fig. 1) agree well with related structures (Harrison *et al.*, 2006; Palani *et al.*, 2006) and literature values (Allen *et al.*, 1987). All non-H atoms   lie in a common plane (r.m.s. deviation 0.029Å) .

The crystal packing is stabilized by weak intermolecular C - H···π [C17—H17C···*Cg*1 (1 - *x*, -*y*, 1 - *z*), H17C···*Cg*1 = 2.68 Å, C18—H18A··· *Cg*2 (1 + *x*, *y*, *z*), H18A···*Cg*1 = 2.75 Å; *Cg*1 and *Cg*2 are the centroid of rings defined by atoms C7/C8/C9/C14/C15/C16 and C1—C6, respectively) interactions. No significant intra- and intermolecular hydrogen bonds are observed.

### Experimental

To a solution of diethyl 2-((2-(bromomethyl)banzo[*b*]thiophen-3-yl) methylene)malonate (0.35 g, 0.88 mmol) in dry 1,2-DCE (15 ml), ZnBr_2_ (0.39 g, 1.73 mmol) and *m*-xylene (0.13 ml, 1.03 mmol), were added. The reaction mixture was then refluxed for 2 h under N_2_ atmosphere. It was then poured over ice-water (50 ml) containing 2 ml of conc.HCl, extracted with chloroform (3 *X* 10 ml) and dried (Na_2_SO_4_). The removal of solvent followed by flash column chromatographic purification (silica gel, 230–420 mesh, n-hexane/ethyl acetate 99:1) afforded 1,3-dimethylbenzo[2,3-*b*] dibenzothiophene as a colourless crystal.

### Refinement

H atoms were positioned geometrically and refined using a riding model with C—H = 0.93 Å and *U*_iso_(H) = 1.2Ueq(C) for aromatic H atoms and C—H = 0.96 Å and *U*_iso_(H) = 1.5Ueq(C) for methyl H atoms.

### Crystal data

**Table:**

C_18_H_14_S	*F*(000) = 552
*M**_r_* = 262.35	*D*_x_ = 1.315 Mg m^−^^3^
Monoclinic, *P*2_1_/*n*	Mo *K*α radiation, λ = 0.71073 Å
Hall symbol: -P 2yn	Cell parameters from 6370 reflections
*a* = 10.0219 (3) Å	θ = 2.1–27.4°
*b* = 5.8692 (5) Å	µ = 0.23 mm^−^^1^
*c* = 22.8554 (5) Å	*T* = 295 K
β = 99.787 (1)°	Block, colourless
*V* = 1324.80 (12) Å^3^	0.26 × 0.20 × 0.18 mm
*Z* = 4	

### Data collection

**Table:**

Bruker Kappa APEXII diffractometer	3030 independent reflections
Radiation source: fine-focus sealed tube	2574 reflections with *I* > 2σ(*I*)
graphite	*R*_int_ = 0.029
ω and φ scans	θ_max_ = 27.5°, θ_min_ = 1.8°
Absorption correction: multi-scan (*SADABS*; Sheldrick, 1996)	*h* = −13→13
*T*_min_ = 0.944, *T*_max_ = 0.961	*k* = −7→7
27929 measured reflections	*l* = −29→29

### Refinement

**Table:**

Refinement on *F*^2^	Primary atom site location: structure-invariant direct methods
Least-squares matrix: full	Secondary atom site location: difference Fourier map
*R*[*F*^2^ > 2σ(*F*^2^)] = 0.046	Hydrogen site location: inferred from neighbouring sites
*wR*(*F*^2^) = 0.137	H-atom parameters constrained
*S* = 1.08	*w* = 1/[σ^2^(*F*_o_^2^) + (0.0636*P*)^2^ + 0.5697*P*] where *P* = (*F*_o_^2^ + 2*F*_c_^2^)/3
3030 reflections	(Δ/σ)_max_ < 0.001
174 parameters	Δρ_max_ = 0.26 e Å^−^^3^
0 restraints	Δρ_min_ = −0.29 e Å^−^^3^

### Fractional atomic coordinates and isotropic or equivalent isotropic displacement parameters (Å^2^)

**Table:**

	*x*	*y*	*z*	*U*_iso_*/*U*_eq_	
C1	0.07838 (18)	−0.0783 (3)	0.33166 (8)	0.0456 (4)	
C2	−0.0587 (2)	−0.0829 (4)	0.30694 (10)	0.0567 (5)	
H2	−0.1153	−0.1953	0.3177	0.068*	
C3	−0.1081 (2)	0.0813 (4)	0.26653 (9)	0.0603 (6)	
H3	−0.1993	0.0801	0.2496	0.072*	
C4	−0.0245 (2)	0.2490 (4)	0.25047 (9)	0.0579 (5)	
H4	−0.0599	0.3589	0.2228	0.069*	
C5	0.1110 (2)	0.2546 (3)	0.27511 (8)	0.0494 (5)	
H5	0.1667	0.3678	0.2641	0.059*	
C6	0.16390 (17)	0.0909 (3)	0.31632 (7)	0.0403 (4)	
C7	0.30153 (17)	0.0662 (3)	0.34821 (7)	0.0378 (4)	
C8	0.41378 (18)	0.1971 (3)	0.34603 (7)	0.0408 (4)	
H8	0.4071	0.3228	0.3209	0.049*	
C9	0.53930 (17)	0.1436 (3)	0.38139 (7)	0.0390 (4)	
C10	0.65545 (19)	0.2770 (3)	0.37964 (8)	0.0472 (4)	
H10	0.6490	0.4027	0.3545	0.057*	
C11	0.77693 (19)	0.2268 (3)	0.41376 (9)	0.0480 (4)	
C12	0.78456 (18)	0.0376 (3)	0.45225 (8)	0.0477 (4)	
H12	0.8674	0.0037	0.4757	0.057*	
C13	0.67622 (18)	−0.0979 (3)	0.45659 (8)	0.0419 (4)	
C14	0.54908 (17)	−0.0488 (3)	0.41992 (7)	0.0381 (4)	
C15	0.43329 (18)	−0.1822 (3)	0.42159 (8)	0.0437 (4)	
H15	0.4381	−0.3086	0.4464	0.052*	
C16	0.31376 (18)	−0.1253 (3)	0.38656 (8)	0.0418 (4)	
C17	0.6892 (2)	−0.2914 (4)	0.49988 (9)	0.0523 (5)	
H17A	0.7795	−0.2944	0.5222	0.078*	
H17B	0.6709	−0.4324	0.4787	0.078*	
H17C	0.6256	−0.2714	0.5265	0.078*	
C18	0.9007 (2)	0.3703 (4)	0.41279 (11)	0.0648 (6)	
H18A	0.9598	0.2941	0.3902	0.097*	
H18B	0.9471	0.3935	0.4527	0.097*	
H18C	0.8742	0.5149	0.3949	0.097*	
S1	0.16072 (5)	−0.26890 (9)	0.38419 (3)	0.0592 (2)	

### Atomic displacement parameters (Å^2^)

**Table:**

	*U*^11^	*U*^22^	*U*^33^	*U*^12^	*U*^13^	*U*^23^
C1	0.0419 (9)	0.0449 (10)	0.0474 (9)	−0.0078 (8)	0.0001 (7)	−0.0003 (8)
C2	0.0429 (10)	0.0597 (12)	0.0632 (12)	−0.0153 (9)	−0.0030 (9)	0.0030 (10)
C3	0.0429 (10)	0.0772 (15)	0.0557 (11)	−0.0015 (10)	−0.0060 (8)	−0.0008 (10)
C4	0.0542 (11)	0.0668 (14)	0.0491 (11)	0.0049 (10)	−0.0018 (9)	0.0105 (9)
C5	0.0511 (10)	0.0519 (11)	0.0442 (9)	−0.0012 (8)	0.0053 (8)	0.0069 (8)
C6	0.0406 (9)	0.0429 (9)	0.0367 (8)	−0.0034 (7)	0.0042 (7)	−0.0033 (7)
C7	0.0391 (8)	0.0378 (8)	0.0361 (8)	−0.0034 (7)	0.0054 (6)	−0.0014 (6)
C8	0.0425 (9)	0.0404 (9)	0.0393 (8)	−0.0059 (7)	0.0068 (7)	0.0049 (7)
C9	0.0388 (8)	0.0408 (9)	0.0383 (8)	−0.0050 (7)	0.0089 (6)	−0.0013 (7)
C10	0.0447 (10)	0.0488 (10)	0.0494 (10)	−0.0102 (8)	0.0120 (8)	0.0035 (8)
C11	0.0391 (9)	0.0550 (11)	0.0513 (10)	−0.0108 (8)	0.0116 (8)	−0.0053 (8)
C12	0.0352 (8)	0.0562 (11)	0.0507 (10)	0.0011 (8)	0.0042 (7)	−0.0036 (8)
C13	0.0401 (9)	0.0432 (9)	0.0422 (9)	0.0020 (7)	0.0066 (7)	−0.0026 (7)
C14	0.0387 (8)	0.0371 (9)	0.0386 (8)	−0.0022 (7)	0.0068 (6)	−0.0023 (7)
C15	0.0445 (9)	0.0353 (9)	0.0497 (10)	−0.0051 (7)	0.0036 (7)	0.0050 (7)
C16	0.0417 (9)	0.0364 (9)	0.0461 (9)	−0.0101 (7)	0.0036 (7)	0.0007 (7)
C17	0.0486 (11)	0.0520 (11)	0.0544 (11)	0.0057 (9)	0.0033 (8)	0.0060 (9)
C18	0.0422 (10)	0.0745 (15)	0.0786 (15)	−0.0188 (10)	0.0127 (10)	−0.0002 (12)
S1	0.0460 (3)	0.0503 (3)	0.0749 (4)	−0.0193 (2)	−0.0080 (2)	0.0180 (2)

### Geometric parameters (Å, °)

**Table:**

C1—C2	1.394 (3)	C10—C11	1.362 (3)
C1—C6	1.395 (3)	C10—H10	0.9300
C1—S1	1.7425 (19)	C11—C12	1.411 (3)
C2—C3	1.368 (3)	C11—C18	1.502 (3)
C2—H2	0.9300	C12—C13	1.363 (3)
C3—C4	1.382 (3)	C12—H12	0.9300
C3—H3	0.9300	C13—C14	1.430 (2)
C4—C5	1.379 (3)	C13—C17	1.497 (3)
C4—H4	0.9300	C14—C15	1.406 (2)
C5—C6	1.386 (3)	C15—C16	1.364 (2)
C5—H5	0.9300	C15—H15	0.9300
C6—C7	1.454 (2)	C16—S1	1.7428 (17)
C7—C8	1.370 (2)	C17—H17A	0.9600
C7—C16	1.418 (2)	C17—H17B	0.9600
C8—C9	1.410 (2)	C17—H17C	0.9600
C8—H8	0.9300	C18—H18A	0.9600
C9—C10	1.409 (2)	C18—H18B	0.9600
C9—C14	1.425 (2)	C18—H18C	0.9600
			
C2—C1—C6	121.14 (18)	C10—C11—C18	122.00 (19)
C2—C1—S1	125.84 (16)	C12—C11—C18	119.55 (19)
C6—C1—S1	113.01 (13)	C13—C12—C11	123.04 (17)
C3—C2—C1	118.62 (19)	C13—C12—H12	118.5
C3—C2—H2	120.7	C11—C12—H12	118.5
C1—C2—H2	120.7	C12—C13—C14	118.76 (16)
C2—C3—C4	120.96 (19)	C12—C13—C17	120.64 (17)
C2—C3—H3	119.5	C14—C13—C17	120.59 (16)
C4—C3—H3	119.5	C15—C14—C9	119.19 (16)
C5—C4—C3	120.53 (19)	C15—C14—C13	121.95 (16)
C5—C4—H4	119.7	C9—C14—C13	118.85 (15)
C3—C4—H4	119.7	C16—C15—C14	119.62 (16)
C4—C5—C6	119.81 (19)	C16—C15—H15	120.2
C4—C5—H5	120.1	C14—C15—H15	120.2
C6—C5—H5	120.1	C15—C16—C7	122.13 (16)
C5—C6—C1	118.93 (16)	C15—C16—S1	125.46 (14)
C5—C6—C7	129.09 (17)	C7—C16—S1	112.39 (13)
C1—C6—C7	111.98 (15)	C13—C17—H17A	109.5
C8—C7—C16	118.80 (15)	C13—C17—H17B	109.5
C8—C7—C6	129.86 (16)	H17A—C17—H17B	109.5
C16—C7—C6	111.34 (15)	C13—C17—H17C	109.5
C7—C8—C9	120.82 (16)	H17A—C17—H17C	109.5
C7—C8—H8	119.6	H17B—C17—H17C	109.5
C9—C8—H8	119.6	C11—C18—H18A	109.5
C10—C9—C8	121.39 (16)	C11—C18—H18B	109.5
C10—C9—C14	119.18 (16)	H18A—C18—H18B	109.5
C8—C9—C14	119.43 (15)	C11—C18—H18C	109.5
C11—C10—C9	121.72 (18)	H18A—C18—H18C	109.5
C11—C10—H10	119.1	H18B—C18—H18C	109.5
C9—C10—H10	119.1	C1—S1—C16	91.28 (9)
C10—C11—C12	118.42 (17)		
			
C6—C1—C2—C3	0.7 (3)	C18—C11—C12—C13	178.60 (19)
S1—C1—C2—C3	179.28 (17)	C11—C12—C13—C14	0.9 (3)
C1—C2—C3—C4	−0.1 (3)	C11—C12—C13—C17	−177.74 (17)
C2—C3—C4—C5	−0.2 (4)	C10—C9—C14—C15	−179.87 (16)
C3—C4—C5—C6	0.0 (3)	C8—C9—C14—C15	0.4 (3)
C4—C5—C6—C1	0.6 (3)	C10—C9—C14—C13	1.3 (2)
C4—C5—C6—C7	−179.00 (19)	C8—C9—C14—C13	−178.50 (16)
C2—C1—C6—C5	−0.9 (3)	C12—C13—C14—C15	179.42 (17)
S1—C1—C6—C5	−179.67 (14)	C17—C13—C14—C15	−1.9 (3)
C2—C1—C6—C7	178.73 (18)	C12—C13—C14—C9	−1.7 (3)
S1—C1—C6—C7	0.0 (2)	C17—C13—C14—C9	176.94 (16)
C5—C6—C7—C8	0.2 (3)	C9—C14—C15—C16	−0.2 (3)
C1—C6—C7—C8	−179.43 (18)	C13—C14—C15—C16	178.66 (17)
C5—C6—C7—C16	−179.98 (18)	C14—C15—C16—C7	−0.3 (3)
C1—C6—C7—C16	0.4 (2)	C14—C15—C16—S1	−178.84 (14)
C16—C7—C8—C9	−0.3 (3)	C8—C7—C16—C15	0.5 (3)
C6—C7—C8—C9	179.59 (17)	C6—C7—C16—C15	−179.40 (17)
C7—C8—C9—C10	−179.90 (16)	C8—C7—C16—S1	179.24 (13)
C7—C8—C9—C14	−0.1 (3)	C6—C7—C16—S1	−0.65 (19)
C8—C9—C10—C11	179.83 (18)	C2—C1—S1—C16	−179.0 (2)
C14—C9—C10—C11	0.1 (3)	C6—C1—S1—C16	−0.29 (15)
C9—C10—C11—C12	−0.9 (3)	C15—C16—S1—C1	179.24 (18)
C9—C10—C11—C18	−179.07 (19)	C7—C16—S1—C1	0.54 (14)
C10—C11—C12—C13	0.4 (3)		

### Hydrogen-bond geometry (Å, °)

**Table:**

*D*—H···*A*	*D*—H	H···*A*	*D*···*A*	*D*—H···*A*
C17—H17C···Cg1^i^	0.96	2.68	3.486 (2)	142
C18—H18A···Cg2^ii^	0.96	2.75	3.649 (3)	155

Symmetry codes: (i) −*x*+1, −*y*, −*z*+1; (ii) *x*+1, *y*, *z*.
